# Methylation-sensitive linking libraries enhance gene-enriched sequencing of complex genomes and map DNA methylation domains

**DOI:** 10.1186/1471-2164-9-621

**Published:** 2008-12-19

**Authors:** William Nelson, Meizhong Luo, Jianxin Ma, Matt Estep, James Estill, Ruifeng He, Jayson Talag, Nicholas Sisneros, David Kudrna, HyeRan Kim, Jetty SS Ammiraju, Kristi Collura, Arvind K Bharti, Joachim Messing, Rod A Wing, Phillip SanMiguel, Jeffrey L Bennetzen, Carol Soderlund

**Affiliations:** 1 Arizona Genomics Computational Laboratory, BIO5 Institute, University of Arizona, Tucson, Arizona, USA; 2 Arizona Genomics Institute, Department of Plant Sciences, BIO5 Institute, University of Arizona, Tucson, Arizona, USA; 3 Department of Genetics, University of Georgia, Athens, Georgia, USA; 4 Department of Plant Biology, University of Georgia, Athens, Georgia, USA; 5 Plant Genome Initiative at Rutgers, Waksman Institute, Rutgers, The State University of New Jersey, Piscataway, New Jersey, USA; 6 Department of Horticulture and Landscape Architecture, Purdue University, West Lafayette, Indiana, USA; 7 College of Life Sciences and Technology, Huazhong Agricultural University, Wuhan, Hubei 430070, PR China; 8 Department of Agronomy, Purdue University, West Lafayette, Indiana, USA; 9 Department of Microbial & Plant Genomics, University of Minnesota, St. Paul, MN, USA

## Abstract

**Background:**

Many plant genomes are resistant to whole-genome assembly due to an abundance of repetitive sequence, leading to the development of gene-rich sequencing techniques. Two such techniques are hypomethylated partial restriction (HMPR) and methylation spanning linker libraries (MSLL). These libraries differ from other gene-rich datasets in having larger insert sizes, and the MSLL clones are designed to provide reads localized to "epigenetic boundaries" where methylation begins or ends.

**Results:**

A large-scale study in maize generated 40,299 HMPR sequences and 80,723 MSLL sequences, including MSLL clones exceeding 100 kb. The paired end reads of MSLL and HMPR clones were shown to be effective in linking existing gene-rich sequences into scaffolds. In addition, it was shown that the MSLL clones can be used for anchoring these scaffolds to a BAC-based physical map. The MSLL end reads effectively identified epigenetic boundaries, as indicated by their preferential alignment to regions upstream and downstream from annotated genes. The ability to precisely map long stretches of fully methylated DNA sequence is a unique outcome of MSLL analysis, and was also shown to provide evidence for errors in gene identification. MSLL clones were observed to be significantly more repeat-rich in their interiors than in their end reads, confirming the correlation between methylation and retroelement content. Both MSLL and HMPR reads were found to be substantially gene-enriched, with the *Sal*I MSLL libraries being the most highly enriched (31% align to an EST contig), while the HMPR clones exhibited exceptional depletion of repetitive DNA (to ~11%). These two techniques were compared with other gene-enrichment methods, and shown to be complementary.

**Conclusion:**

MSLL technology provides an unparalleled approach for mapping the epigenetic status of repetitive blocks and for identifying sequences mis-identified as genes. Although the types and natures of epigenetic boundaries are barely understood at this time, MSLL technology flags both approximate boundaries and methylated genes that deserve additional investigation. MSLL and HMPR sequences provide a valuable resource for maize genome annotation, and are a uniquely valuable complement to any plant genome sequencing project. In order to make these results fully accessible to the community, a web display was developed that shows the alignment of MSLL, HMPR, and other gene-rich sequences to the BACs; this display is continually updated with the latest ESTs and BAC sequences.

## Background

The nuclear genomes of grass species vary widely in size due to polyploidization and amplification of repeat elements. On the smaller end of the size spectrum lies rice (*Oryza sativa*) whose ~390 Mb genome has been sequenced [1]. Mid-sized genomes such as that of maize (*Zea mays*, 2.4 Gb) present a far greater challenge for sequencing, while large genomes such as bread wheat (*Triticum aestivum*, 17 Gb) will require exceptional approaches. The great differences in genome size are mainly caused by differences in repetitive DNA content, primarily LTR (long terminal repeat) retrotransposons that can comprise more than 50% of a nuclear genome. Because the amplification of these sequences occurred in waves, assembly of contiguous sequence information without a physical map is difficult or impossible for genomes as large as the maize genome [2].

Despite these great variations in genome size, the gene content of these species appears to be about the same per 2 N genome [3]. Therefore, a number of "gene-enriched" sequencing techniques have been proposed with the goal of sequencing primarily the genic regions, while excluding repetitive sequence as much as possible. The oldest of these is EST sequencing, consisting of end reads from cDNA clones. More recently, full length cDNAs have been sequenced in rice [4] and Arabidopsis [5], and a similar project is underway in maize [6]. These are by far the most gene-enriched techniques, albeit with some contamination from transcribed transposable elements (TEs) and other repeats [7]. In addition, these transcription-based techniques automatically eliminate transcriptional pseudogenes, a non-trivial undertaking when analyzing genomic sequence alone. However, EST sequencing also has significant drawbacks, of which the most important is that it is strongly affected by transcriptional biases. Some genes are expressed at high levels per cell or tissue, while others make only a handful of mRNA molecules, and/or are active only in certain tissues or developmental phases. Hence, EST sequencing tends to miss some genes while sequencing others many times over, a costly redundancy. Furthermore, sequencing of expressed products captures only exons and excludes all information about promoter regions, and paralogs may be easily conflated because intronic sequence data are missing.

To overcome these drawbacks, other techniques have been developed that work directly with nuclear DNA. The most generic is high-CoT sequencing (HC), in which sheared DNA is separated based on renaturation time. Repetitive fragments renature faster, allowing them to be removed. This method has been applied in maize and resulted in 6-fold enrichment of genic sequences [8,9]. A second approach, referred to as methyl-filteration (MF), makes use of the special properties of methylation in higher plant genomes. In these genomes, repetitive DNA is generally found to be hypermethylated, while genic regions are hypomethylated, permitting enrichment by cloning into bacteria that do not tolerate some forms of cytosine methylated DNA [10-14]. For the MF technique, DNA is sheared from total genomic DNA and is inserted into a plasmid vector, followed by transformation of the library into an *Escherichia coli *host that will not tolerate clones containing methylated DNA inserts; in maize this technique yielded gene enrichment comparable to the HC reads [8,15]. A third approach employed in maize makes use of the RescueMu transposable element [16], relying on the fact that *Mutator *elements preferentially insert into low-copy-number DNA [17].

A large dataset of HC, MF, and unfiltered (UF) sequences has been produced and assembled to generate the assembled *Zea mays *(AZM) contigs [8,18]. Version 4 of these assemblies was constructed from 450,166 MF, 445,565 HC and 50,866 unfiltered (UF) reads. This dataset contains separate MF-only, HC-only and UF-only assemblies, which are henceforth referred to as the "MF", "HC", and "UF" datasets. The RescueMU assemblies [19], henceforth referred to as "RM", are also available for comparison.

The MF and HC datasets were derived from small-insert clones, with mean length of 2 kb for HC clone inserts and <1.5 kb for MF clone inserts. Small inserts were essential for these techniques, since longer clones will often include repetitive elements along with genes and thereby be excluded; however, the small sizes limit the ability of the read pairs to link contigs into scaffolds. RescueMu-flanking sequences also do not link over a substantial distance, as the reads are adjacent to the insertion site. Assembly of such reads leads to short contigs that result in a gapped alignment along a gene, especially because transposable elements may be present within introns [20] and between core promoters and regulatory elements [21,22]. Although comparison with EST databases and sequenced clones indicates that almost every gene is represented in the assemblies, only about 30% were fully covered by the first ~450,000 MF and HC reads [8,23]. When these reads were compared to 100 random genomic regions of the maize genome, only 29% of the predicted genes were covered over more than 90% of their length [20]. Furthermore, sequences assembled from these gene-enriched sets cannot be easily localized on a physical or genetic map.

To overcome these limitations, two complementary techniques have been proposed. Both of these approaches generate paired end reads from longer clones, allowing them to link contigs generated by the previous methods. Both techniques rely on methylation-sensitive restriction enzymes to cleave nuclear DNA preferentially in genic regions. For methylation-spanning linker libraries (MSLL, [24]), DNA was subjected to complete digestion by restriction enzymes such as *Sal*I or *Hpa*II, and fragments of varying sizes (from 7 kb to >100 kb) were cloned. The relatively long length of these clones allows them to span repetitive regions between genes, thereby linking the genes; it also allows their integration into a BAC-based physical map based on DNA fingerprints. Hypomethylated partial restriction (HMPR, [25]) libraries are similar but utilize only enzymes having 4-bp recognition sequences. Partial digestion was employed, and fragments from 2–4 kb were selected for cloning and end-sequencing. The need for two unmethylated sites in close proximity ensures that these clones often sample low-copy-number sequences, and that they can also provide valuable information for linking contigs into scaffolds.

Pilot studies (in maize) of 751 MSLL sequences [24] and 2112 HMPR sequences [25] demonstrated enrichment of genes (and depletion of retroelements and other repetitive DNAs) equal to or greater than that seen with MF or HC, with HMPR producing the greatest retroelement depletion seen outside of EST libraries. Such pilot studies cannot indicate, however, when such approaches saturate (i.e., lose value due to repetition in the data generated) or how generally useful they can be across a genome that has been largely or fully sequenced.

Although the DNA composition and arrangement in most plant genomes is complex and only narrowly understood, the much greater epigenetic complexity of these genomes is even more mysterious. Both MSLL and HMPR technologies provide full-genome capacity for the discovery of methylated blocks [24,25]. Comprehensive analysis of a genome with MSLL and HMPR will uncover all of the blocks of DNA that are completely methylated, perhaps in a tissue or at different times in development, and these can be studied to find unusual components like methylated genes or unmethylated transposable elements. As with any genomics technology, a comprehensive and high-throughput use of MSLL and HMPR can identify and highlight important components that deserve more detailed study.

The following study reports results of a comprehensive study of MSLL and HMPR sequences in maize. The observations from the pilot studies are confirmed and extended. It is shown that MSLL clones longer than 100 kb may be generated, and that MSLL clones of size 35 kb and higher can be accurately placed on a BAC-based FPC map (fingerprinted contigs, [26,27]). The MSLL clones are found to be particularly valuable for identifying fully methylated DNA blocks that could be discovered by no other technique, thereby allowing the identification of "genes" that are either annotation artifacts or exceptional in their epigenetic status. These valuable resources are made available to all scientists by providing their alignment to sequenced maize BACs as a web-based service [28].

## Results

### Library Production

The details of the production of the MSLL and HMPR libraries are summarized in Table 1. A total of 21,696 HMPR clones of size 2–4 kb were produced and end-sequenced, yielding 40,299 successful reads, with 37,316 (93%) paired. The clones comprised 19 different libraries, built using one of two 4-bp specificity enzymes, *Hpa*II or *Hpy*CH4 IV, and different degrees of partial digestion. A total of 44,617 MSLL clones were produced and end-sequenced, yielding 80,732 successful reads, with 72,984 (90%) paired. The clones comprised 10 different libraries, built using either *Hpa*II (50,686 reads) or *Sal*I (30,046 reads), with insert sizes ranging from 7 kb to over 100 kb. One MSLL library used high-copy plasmid vectors with inserts of 7–12 kb, while the rest used BAC vectors.

**Table 1:** HMPR and MSLL libraries, and alignment to 16,861 BACs.

**Library Data**				***BAC Alignment Data***		
HMPR ZMMB^1^ Library^2^	Enzyme	Digestion	Reads	*Paired Hits*	*Avg Span (kb)*	*Avg %* *Masked*^3^

Ha	HpyCH4 IV	10 U/ug	171	27	3.1	28%
Hb	HpyCH4 IV	0.1 U/ug	181	26	2.7	19%
Hc	HpaII	10 U/ug	734	120	3.5	28%
Hd	HpaII	0.5 U/ug	1482	278	3.5	25%
He	HpaII	0.25 U/ug	5189	913	3.3	27%
Hf	HpaII	0.125 U/ug	10428	1838	3.1	25%
Hg	HpaII	0.05 U/ug	1488	246	3.2	27%
Hh	HpaII	0.025 U/ug	177	19	3.1	19%
Hi	HpaII	0.0125 U/ug	174	22	2.6	18%
Hj	HpaII	0.005 U/ug	177	34	2.9	38%
Hk	HpaII	0.00125 U/ug	183	29	2.4	37%
Hl	HpyCH4 IV	0.5 U/ug	1441	112	2.2	31%
Hm	HpyCH4 IV	0.25 U/ug	2239	239	3.0	36%
Hn	HpyCH4 IV	0.125 U/ug	1492	239	3.0	34%
Ho	HpyCH4 IV	0.05 U/ug	2550	492	1.9	21%
Hp	HpyCH4 IV	0.025 U/ug	8218	1483	3.0	22%
Hq	HpyCH4 IV	0.0125 U/ug	1782	329	2.9	25%
Hr	HpyCH4 IV	0.005 U/ug	1544	262	2.3	23%
Hs	HpyCH4 IV	0.002 U/ug	649	115	2.3	29%
Total			40299	6865	3.0	26%
MSLL ZMMB^1^ Library	Enzyme	Length (kb)	Reads	Paired hits	Avg Span (kb)	Avg % Masked^3^
La	SalI	35–60	7536	93	46	78%
Lb	HpaII	35–60	8733	146	39	82%
Lc	HpaII	20–35	9639	483	26	78%
Ld	SalI	20–35	10916	469	24	56%
Le	HpaII	12–20	8920	630	15	65%
Lf	SalI	60–100	5436	10	80	78%
Lh	SalI	>100	6158	0	n/a	n/a
Li	HpaII	60–100	8495	21	70	86%
Lj	HpaII	>100	7715	0	n/a	n/a
Lz	HpaII	7–12	7184	807	11	55%
Total – Hpa			50,686	2087		66%
Total – Sal			30,046	572		60%
Total			80,732	2659		65%

^1 ^The ZMMB libraries followed by the indicated suffix (e.g. ZMMBLj) can be ordered from the Arizona Genomics Institute [43].

^2 ^HMPR clones have size ranges of 2–4 kb.

^3 ^The percent of sequence masked within the spanned BAC regions, as determined by RepeatMasker against the TIGR maize repeat database.

The sequences have been deposited in Genbank with the following accession numbers: MSLL; CW003489–010484, CW010655–020123, DX824179–887735; HMPR; CL983298–999029, CW000001–002948, CW509394–510127, ER990272–ER999999, ET000001–ET010187.

The intent of the multiple libraries was to cover the maize genome thoroughly, as each library is expected to sample a somewhat different space. The MSLL clones were complete digests, so there should not be multiple clones of different lengths covering the same region, for libraries having the same cloning enzyme. The different *Sal*I and *Hpa*II libraries should therefore be mutually exclusive, both in the genome regions they cover and (aside from repetitive sequence) in their end reads. Between the *Sal*I and *Hpa*II library sets there will be overlap in the genomic regions covered, but the end-read sets are still expected to be disjoint, because the 4-bp recognition pattern of *Hpa*II causes the cut site to be much closer, on average, to the starting point of the methylated region (the "epigenetic boundary" [24]). Because of this significant difference in the expected placement of the end reads, separate results are provided for MSLL-*Sal *and MSLL-*Hpa*.

In the case of HMPR, the size selection combined with the differing digestion levels (Table 1) should ensure that each library samples regions with different average densities of cloning enzyme restriction sites [25], and the two different restriction enzymes were used to target regions of maize with greater and lesser GC content (*Hpa*II has recognition sequence 5'-CCGG-3', while *Hpy*CH4 IV recognizes 5'-ACGT-3'). The presence of 5-methylation at any of the cytosines in these restriction sites will fully block digestion. Considerable overlap was found between the HMPR read sets, and overall very similar properties were observed for the libraries, so the HMPR results are not usually reported separately by cloning enzyme.

The MSLL and HMPR reads were aligned to sequenced BACs to verify the size of the clones and to investigate the content of the internal sequence. A total of 16,861 maize BACs were downloaded from Genbank, of which 16,704 had MSLL or HMPR alignments. Of these BACs, 357 had ordered sequence contigs. The alignments were required to be paired, with stringent filtering applied to reduce false-positive alignments from the dataset (see Methods). Paired alignments were found for 6865 HMPR clones and 2659 MSLL clones, including representatives from all but two of the libraries. From the alignments, the clone lengths were estimated and found to be in accordance with expectations (Table 1).

The repeat content of the BAC sequences was characterized by masking them against the TIGR v4.0 repeat database ([18]; see Methods). Having done this, the BAC alignments were used to estimate the repeat content of the whole MSLL and HMPR clones (Table 1). For the HMPR clones, the percentage of masking was relatively low, from 18% to 36% with an average of 26%, while for MSLL libraries the percentage varied from 55% to 86% with an average of 65%.

Organelle content of the sequences was also analyzed and used to develop an estimate of chimerism in the MSLL and HMPR clone libraries. Organellar sequence was found in 3.4% of MSLL clones and 6.9% of HMPR clones. If chimerism is present in the libraries, some clones should exhibit organellar sequence on one end and genomic sequence on the other, with the ratio of single to paired organellar sequences providing a measure of the chimeric fraction. Applying this test (and accounting for the possibility of organelle-related DNA in the genome; [29]) led to an estimate of 1.3% chimerism in the MSLL and 0.15% in the HMPR (see Methods).

By randomization of the data and sampling with replacement, the last 1000 sequence reads were analyzed for new sequence discovery. HMPR yielded 438 unique sequences, while MSLL yielded 127 novel sequences in this last 1000 after 40,000 and 80,000 reads were analyzed, respectively. Both of these numbers are actually under-estimates of the true novelty of sequence discovery, because the same sequence found two or more times was often caused by different copies of the same repeat. Hence, for MSLL, the true rate of novel sequence discovery may be 3-fold higher and as much as 25% higher for HMPR. Even unadjusted, the level of novel sequence discovery for HMPR after this many clones are analyzed is exceptional, far exceeding rates of novel sequence discovery by EST analysis, for instance [8]. These results also indicate that these two technologies were far from saturated at the current level of analysis in the maize genome.

Since MSLL clones resulted from a complete digestion, they should uniquely cover their genomic region of origin. Therefore, when comparing paired end reads from two clones, either both ends or neither end should match. To test this, the ratio of clone pairs matching on both ends to those matching on one or both ends was calculated (Methods). A total of 21972 MSLL clone pairs matching on at least one end were identified, of which 9687 (44%) matched on both ends. The effect was relatively constant across the different MSLL libraries, with nine of the ten libraries having pairing fractions between 45% and 63%, and only one (Lb) being substantially different (23%).

### Coverage of gene space

The end-sequences of HMPR and MSLL clones preferentially tag genes and therefore constitute another form of gene-enriched sequencing [24,25]. It is important to characterize the degree of the enrichment, and in what ways the gene space coverage of the various techniques are different. For example, if the MSLL sequences cover regions distinct from the MF and HC sequences, then their effectiveness as linkers will be reduced; conversely, if the HMPR cover unique regions, then they may be effective additions to a gene-rich sequencing strategy.

The HMPR, MSLL, MF, HC, UF, and RM sequences were compared to maize EST contigs [6] and the TIGR maize repeat database [18]. As shown in Table 2, the MSLL-*Sal *sequences were quite enriched for genic (EST) sequences, while the HMPR were similar to the HC, MF, and RM sequences, and the MSLL-*Hpa *were less enriched. HMPR sequences were also highly depleted in retroelement sequence (as identified by RepeatMasker; see Methods), being slightly more depleted than the HC, and only the RM exhibited greater depletion.

**Table 2:** Comparison of different gene enrichment and unfiltered methods, showing percentages of sequence masked by repeats in various categories of the TIGR v4.0 maize repeat database, as well as percentages of sequences having similarity to a maize EST contig.

	**MSLL-Sal**	**MSLL-Hpa**	**HMPR**	**MF**	**HC**	**RM**	**UF**
Sequences^1^	30,046	50,686	40,299	133,806	172,600	191,715	49,364
Avg. size (bp)	671	698	932	1172	1094	337	748
Retro	20%	20%	11%	26%	12%	4%	64%
Transposon	0.6%	0.9%	0.7%	0.5%	0.7%	0.3%	0.6%
MITE	0.6%	1.3%	1.0%	0.4%	0.7%	0.6%	0.2%
Centromere	0.2%	0.9%	0.2%	0.4%	0.09%	0.02%	0.8%
Telomere	0.001%	0.03%	0.08%	0.02%	0.02%	0.02%	0.01%
Ribosomal	0.4%	1.5%	1.3%	0.2%	0.1%	0.1%	2.1%
Unknown	14%	19%	13%	9%	14%	7%	14%
Total repeat	37%	42%	28%	38%	27%	12%	82%
EST Contigs	31%	16%	22%	25%	21%	22%	7%

^1 ^The MF, HC sequences are contigs of reads; all other types are direct reads.

Known MITE transposons are a quantitatively minor component of the maize genome, but were quite enriched in the MSLL and HMPR end-sequences compared to the UF control. MSLL-*Hpa *reads were particularly rich in these predicted MITEs (1.3%), while the MSLL-*Sal *clones were rather less enriched (0.6%).

**Figure 1:**
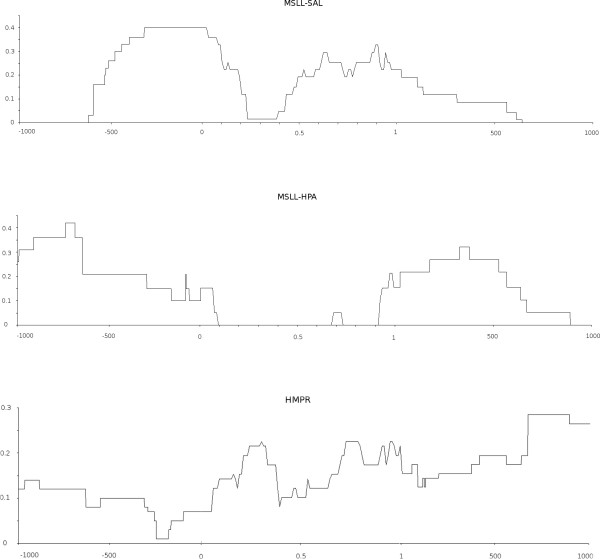
**Alignment of MSLL-*Sal*, MSLL-*Hpa *and HMPR sequences to 105 curated gene sequences, which included at least 1 kb of upstream and downstream intergenic sequence (see text)**. The horizontal scale in the upstream and downstream regions is basepairs; in the gene interior, it is fractional distance along the gene length. The vertical scale indicates the fraction of gene-aligning sequences which cover the gene region in question.

To investigate the coverage of genic regions by HMPR and MSLL sequences, 151 annotated maize gene sequences were employed, each consisting of one gene along with introns and varying amounts of upstream (5') and downstream (3') intergenic sequence (Brad Barbazuk, pers. comm.). A total of 75 HMPR sequences (including 19 paired) and 63 MSLL (with 2 paired) could be aligned to these genes (see Methods). The positional distributions of the gene alignments are shown in Figure 1, which was created using a subset of 105 genes for which at least 1 kb of up- and downstream sequence was present in the annotated sequence set. Each graph covers the 1 kb upstream, the gene interior, and the 1 kb downstream regions. This subset included 56 HMPR alignments to 28 different genes, 19 MSLL-*Hpa *alignments to 12 different genes, and 29 MSLL-*Sal *alignments to 18 different genes. Both classes of MSLL alignments exhibit strongly bimodal distributions, with 5' and 3' peaks and a trough in the gene interior. The pattern was most pronounced for the MSLL-*Hpa *inserts, for which the distribution was almost entirely concentrated in the 5' and 3' intergenic regions, while the MSLL-*Sal *end reads extend into the gene itself. The HMPR alignments exhibited a more even distribution.

**Figure 2:**
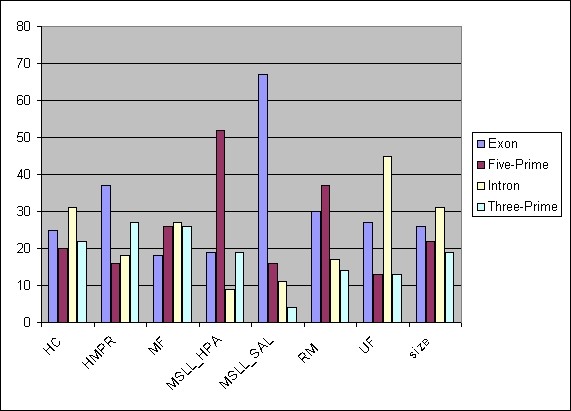
**Starting point location of alignments of sequence types to gene regions**. MSLL, HMPR, MF, HC, RM, and UF sequences were aligned to 151 curated gene sequences (see text). Bars indicate the percentage of the alignments for each sequence type for which the initial base was located in the indicated gene region (exon, intron, 5' intergenic, 3' intergenic). The "size" field shows the total amount of sequence of each type.

Also interesting are the starting locations of the reads for the various sequence types. Figure 2 shows the percentage of alignments for each type that begin in one of four different gene regions, either exons, introns, upstream DNA, or downstream DNA. (The full set of 151 curated genes was used for this plot.) The MSLL-*Sal *sequences were the most likely (67%) to begin within an exon, and were the least likely (4%) to begin within the 3' region. In contrast, the MSLL-*Hpa *sequences were the most likely to begin in the 5' region (52%), and were among the least likely to begin within either an exon (19%) or an intron (9%). HMPR reads were the second most likely to begin within an exon (37%). The MSLL end reads were seen to be by far the most unevenly distributed class.

The MSLL sequences are expected to begin in unmethylated regions, and (since they result from complete digestion) to be fully methylated in the interior. Given the association between repetitive elements and methylation, it is expected that repetitive elements should be less frequent near the start of these reads (i.e., the end of the clone) as compared to the end of the reads (i.e., the clone interior). Also, this effect is predicted to be most pronounced for the *Hpa*II clones, whose ends lie closer to the epigenetic boundary. Analysis of the repeat-masked reads bears this out: MSLL-*Hpa *reads were 2.5 times as likely to be masked in the last 100 bp compared to the first 100 bp, while MSLL-*Sal *reads were 50% more likely, and UF control sequences were equally likely to be masked at either end. Both varieties of MSLL clone were much more likely (77%) to be masked within their interiors (Table 1) than on their end reads (37–42%; Table 2).

### Linking of gene-rich contigs and placement on the FPC map

MSLL and HMPR sequences were aligned to the TIGR AZM v5.0 contigs (assemblies of both MF and HC reads; [18]) as described in Methods. In all, 15,730 (39%) of the HMPR and 24,217 (30%) of the MSLL reads could be aligned to 31,427 AZM contigs (11% of the total). Links between two different AZM contigs were made by 8230 HMPR and 7763 MSLL clones. As expected given their small size, a significant number of HMPR reads (2858) had both ends within a single AZM. The links were used to construct scaffolds of which 256 contained 4 AZMs, 52 contained 5 AZMs, and 9 contained >5 AZMs (with a maximum of 10). The individual linking pairs were further checked, where possible, against the sequenced maize BACs, with 85% of 5239 testable links being verified.

In order to place the scaffolds on a physical map, the MSLL clones were fingerprinted and integrated into the maize HICF FPC map [30]. A total of 1920 clones were fingerprinted using the HICF methodology of [30], where 1152 were from 35–60 kb libraries (La, Lb), 384 were from a 60–100 kb library (Lf), and 384 were from a >100 kb library (Lh). A total of 1022 successful fingerprints were generated, of which 923 (90%) were placed onto the map, in approximate agreement with the 83% estimated genome coverage achieved by the map [30]. Of the 923 placed clones, 651 were in the 35–60 kb group, for a placement rate of 87%, which is significant as it demonstrates that clones that are much shorter than the original clones in the map (~150 kb) may be located on an HICF FPC map.

Placements were checked for accuracy using the alignments to the sequenced BACs, as the location of the MSLL inserts on the FPC map should correspond to the location of the BAC that it aligned to. Of the 35–60 kb MSLL, 124 placements could be checked and 123 were verified, while 8 placements could be checked for the >60 kb range, and all were verified.

### MSLL clone positions across the largest current contiguous assembly in maize

**Figure 3:**
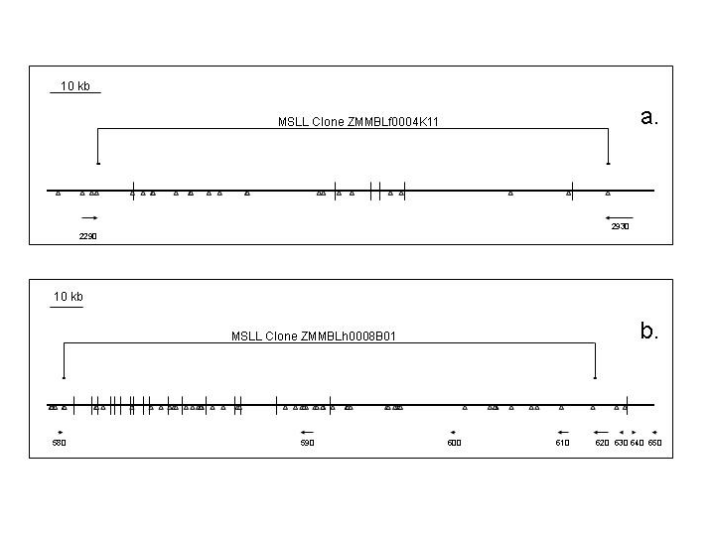
**Example alignments of MSLL BACs with maize genomic assemblies**. (a) routine observation, with the genes shown by arrows and the areas between the genome primarily comprised of LTR retrotransposons and a few other repeats. (b) unusual alignment, where MSLL ends flank apparently methylated genes. Predicted genes are shown by arrows, with the size and orientation indicating the predicted size and transcriptional orientation of the candidate gene. Each vertical lines indicates a gap in the sequence assembly, while the triangles indicate sites for the restriction enzyme *Sal*I, which was used to generate the two BACs shown. The genomic sequence scaffolds depicted are from Bruggmann and coworkers [31].

Bruggmann and coworkers [31] sequenced and assembled/annotated 7.8 Mb and 6.6 Mb regions on maize chromosomes 1 and 9, respectively, from inbred B73. When MSLL clone data were mapped across these regions, most MSLL BACs with paired end data were found to link genes that were separated by a large block annotated as mostly repetitive DNA (Fig. 3a). This standard result indicates that all of these intervening repeats are 100% methylated at the restriction sites used for BAC construction. In rarer cases (Fig. 3b), genes were annotated in the area between the MSLL BAC ends. These may be exceptional genes that are 100% DNA methylated for the BAC construction enzyme sites, or more likely, are genome annotation errors. In the case shown in Fig. 3b, three genes (590, 600 and 610) are predicted to be inside a fully methylated region. Although this could be caused by the methylation of these genes, it could also be a result of mis-identification of low-copy-number transposons as genes [3] or assembly of the contigs into an inaccurate scaffold. In any of these three cases, the region is now identified as one that deserves further inspection. It should be noted that the epigenetic boundaries identified by MSLL clones need not always be between unmethylated genic regions and methylated regions full of transposable elements. In some cases, where genes are extensively or fully methylated, an epigenetic boundary can be fully genic and would be uncovered by MSLL or HMPR clones that indicate an absence of digestibility within that gene.

### Maize gene-searching and location web site

**Figure 4:**
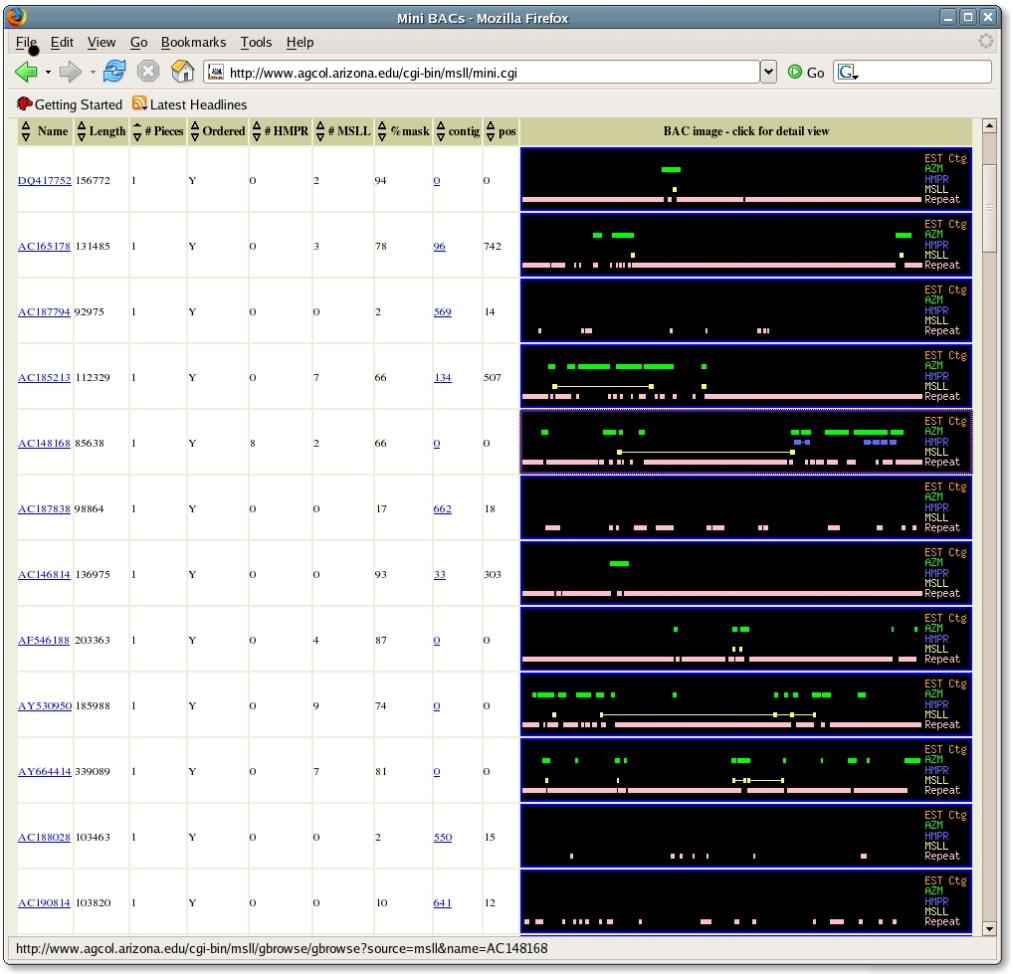
**The maize mini-BAC page **[28]. The Name entries link to the Genbank record and the Contig entries link to the WebFPC contig display [48] to show the position of the clone. Clicking on a BAC icon displays a Genome Browser for the BAC, with additional tracks for RM, HC, and MF sequences.

Though all MSLL and HMPR sequences are available from Genbank, their utility is greatly augmented for the public if they are available for search and location through a specialized web site. To this end, they have been made available online [28]. The location of the MSLL, HMPR, AZMs, EST Contigs and repeats along each BAC is shown in the 'mini-BAC' table (see Fig. 4), which provides an easy way to locate the gene-rich regions on the BACs. Selecting a BAC displays its details in the genome browser (GBrowse; [32]), and a versatile Java display is also provided. The BAC genome browser view also shows RM insertion sites, TIGR repeats, and Fgenesh predictions [33]. This site is updated with an automatic system that downloads new or modified BACs from Genbank. Additionally, a user-supplied sequence can be searched against any of the four types of sequences, with the alignment display linking in turn to the sequence and location on the BAC.

## Discussion

This project was initiated for two reasons. First, to test the degree to which MSLL and HMPR technologies could enhance an efficient gene-enrichment approach to the characterization of large and complex genomes like those found in flowering plants and, second, to determine how efficiently epigenetic information could be added to a complex genomic sequence analysis. Maize was chosen as the test organism because of the difficulty in assembling contiguous sequences from its 2.4 Gb genome [34,35]; because a large database of MF and HC sequences exists for these species [8]; and because many BAC sequences have been and are currently being generated ([20,31,36]). In the process of characterizing the efficacy of MSLL and HMPR for aiding the finishing and linkage of shotgun sequences, the results provided additional insights into the structure and epigenetic status of the maize genome.

### Comprehensive coverage and possible artifacts in the HMPR or MSLL data

Very few candidate chimerics were detected in the more than 36,000 MSLL clones with paired end reads. Only 150 clones exhibited an organellar DNA sequence at one end and non-organellar DNA at the other, and most of these were observed to be likely cases of organellar DNA inserted into the nuclear genome (Methods). Given the incomplete state of the maize genome sequence, it is quite possible that all of these apparent chimerics are actually cases of nuclear genomic regions with inserted organellar DNA. Apparent organellar chimerics in the HMPR data were even rarer, probably because organellar DNA insertions into the nuclear genome are likely to be extensively cytosine methylated, and nearly all the candidates were found to be likely cases of organellar insertions in the nuclear genome.

Among MSLL clones, which are the products of a complete digest, another possible indication of chimeric clones would be those that share the same sequence at one end but a different sequence at the other. Among 21972 apparently redundant MSLL clones, 44% were found to have the same sequence at both ends and 56% were found to have the same sequence at one end and a different sequence at the other. However, given the near-zero chimeric rate predicted by the organellar clones, it seems likely that most of this 56% must be caused by some other phenomenon than chimerism. Using a newly identified low-copy-number database for maize (R. Baucom and J. Bennetzen, unpub. obs,) to scan these data, many of the clones with one different end sequence were found to be cases where the shared end sequence was in fact repetitive but not masked using existing repeat databases. Hence, the "shared" ends were not truly shared, but were different copies of the same low-copy-number repeat, and these low-copy-number repeats appear commonly to be present in an unmethylated state. The partially-matching MSLL clones that would remain, if any, after a full masking by maize repeats, could indicate a lack of homogeneity in epigenetic status within the tissues sampled for DNA preparation.

The number of clones needed for complete genome coverage is expected to vary widely across different MSLL and HMPR libraries. For instance, *Sal*I is predicted to have about 60,000 sites in the maize genome, with about 16-fold more sites for *Hpa*II or *Hpy*CH4 IV, although the majority of these sites will be methylated. How sites are clustered, whether they are more or less abundant in the major repeats and several other issues can also affect the outcomes of MSLL and HMPR experiments. Taking all of these issues into account, the major technical significance of this question is clear. Moreover, it is also clear that the answers could only be provided when large-scale analyses were conducted like the one described herein.

Because the number of gene islands is constant within a specific DNA source (i.e, the inbred B73), the actual number of MSLL-*Sal *and MSLL-*Hpa *clones needed to find all of these islands would be approximately the same (excepting the fact that many islands may completely lack an *Sal*I site and a few may lack any *Hpa*II site). However, it would be predicted that deeper sequencing of larger size clones would be needed, on average, for MSLL-*Sal *compared to MSLL-*Hpa*. After 40,000 MSLL BACs had generated paired end sequence data, >12% of the last 1000 analyzed were still yielding unique linkages, compared to 33% in the 1000 MSLL clones analyzed. Hence, this technology was far from saturated, and analysis of another 40,000 MSLL BACs would have been appropriate.

The HMPR libraries generated >43% unique sequences in the last 1000 of 40,000 generated reads (compared to 78% unique in the first 1000). Hence, the HMPR approach was far from saturation. If the maize genome is approximately 20% unmethylated and genic [8], then this 480 Mb is expected to have about 2–3 million digestible sites each for *Hpa*II and *Hpy*CH4 IV, so as many as a million reads from HMPR libraries would be appropriate to fully utilize this exceptional gene discovery technology on the maize genome. Of course, any genes that are highly methylated in the tissues analyzed may not be enriched by HMPR technology, depending on methylated block size and the length of cloned fragments, but their ends might be uncovered by the MSLL approach, especially if small fragments are cloned.

### Maize genome structure and the distribution of DNA methylation

The use of MSLL and HMPR libraries further confirmed, as previously observed by many laboratories, that most of the maize genome is highly cytosine 5-methylated at 5'-CG-3' and 5'-CNG-3' residues, and that this DNA modification is rarer in genes than in the LTR retrotransposons that make up the majority (>60%) of the maize genome [20]. This is illustrated both by the HMPR libraries, which have substantial enrichment for genic sequences coupled with a very low frequency of LTR retrotransposon sequences, and by the MSLL libraries, for which the incidence of retroelements increases greatly within the methylated interior region of the clone as compared to the end sequences, which are at least terminally unmethylated (Table 1).

A significant fraction (23%) of the DNA inside the MSLL clones was found not to match known LTR retrotransposons or other abundant repeats (Table 1). Such non-repetitive, non-genic DNA was noted previously [20], and the present work extends this observation to show that much of this DNA is also cytosine-methylated. The nature of this DNA is unknown, but it is possible that it may be either currently unannotated LTR retrotransposons that are low in copy number, or degraded fragments of such elements [37]. In support of this interpretation, recent studies (R. Baucom and J. Bennetzen, unpub. res.) have discovered dozens of new families of LTR retrotransposons that make up a good portion of the maize genome, but have low copy numbers (less than 100 per genome) that have led to their current absence from any repeat-masking database.

The enrichment of MITEs in MSLL end sequences was expected because previous studies have shown that MITEs preferentially accumulate near genes, especially near the proposed insulator elements at the 5' ends of genes that are predicted to protect genes from spreading inactivation by surrounding heterochromatin [38]. The greater enrichment of MITEs within the end sequences of MSLL clones using *Hpa*II as the generating enzyme was also expected since *Sal*I, a 6-bp recognition enzyme, should digest on average ~2 kb from the epigenetic boundary, giving rise to an end read that would not often reach the boundary. This also explains the greater gene discovery potential of *Sal*I MSLL clones, because the recognizable coding exons of genes are usually several hundred bp to a few kb away from the epigenetic boundary where *Sal*I cuts.

The overall pattern of MSLL end read alignments to genes (Fig. 1) was as expected, with 5' and 3' peaks and a central minimum reflecting the relationship of MSLL ends to epigenetic boundaries, and with both of these characteristics more pronounced for the *Hpa*II clones relative to the *Sal*I clones. This indicates that a combination of *Sal*I and *Hpa*II MSLL clones would be most effective in extending gene sequences to the epigenetic boundary and then linking them across methylated regions.

MSLL clones also provide a unique window into the DNA methylation status of large stretches of the maize genome. Because they are derived from complete digestions with methylation-sensitive restriction enzymes, the end sequences of a MSLL BAC prove that the DNA between those ends was completely methylated at all sites for that restriction enzyme in the tissue that was the source of the DNA. Such techniques as hybridization of immunoprecipated chromatin or DNA [39] and bisulfite sequencing [40] are able to detect altered epigenetic states in a genomic region, but they are unable to easily or efficiently deal with specific repeats in highly repetitive regions. It would not be a major challenge to utilize the MSLL technique across tissue types to see if and when DNA methylation patterns change across whole genomic regions during development or under some environmental conditions. In addition, when predicted genes are found inside a methylated block, this conflicts with the general observation that plant genes are unmethylated regardless of the tissue analyzed [11]. Thus, these genes may be particularly interesting targets for the study of epigenetic regulation of genes [41] and/or candidates that should be further investigated to see if they are actually artifacts of incorrect genome annotation [3].

### Efficient genome sequencing with gene enrichment techniques

Gene enrichment approaches to genome characterization are designed to provide the maximum amount of informative sequence in a short timeframe and at low cost. Ideally the sequence set would include (1) the sequences of all genes (including their promoters, introns, and UTRs) and (2) the locations of all genes on physical and genetic maps. The highly complementary HC and MF approaches have been proven to tag most maize genes with very high efficiency at least with one read [8,20,23]. However, comparison of HC and MF data to 100 random regions of the maize genome indicates an under-representation of sequences flanking the coding region that contain regulatory elements [20]. The results herein show that MSLL reads, particular from the *Hpa*II libraries, drive sequencing into the 5' and 3' flanking regions (Fig. 1). In addition, both HMPR and MSLL clones were successful in linking AZM contigs into larger scaffolds, even at the low coverage attained in this project. This is essential for spanning whole gene regions, given that regulatory and coding sequences may be separated by the insertion of LTR retrotransposons, and that introns may also be enlarged due to the presence of repetitive elements [20-22].

The results also show that MSLL clones, uniquely among gene-enriched sequence types, can be fingerprinted and placed on the physical map by the now-routine HICF technology. Since their end sequences will often overlap AZMs, DNA markers used for genetic mapping, and other gene-rich sequence assemblies, they can provide indispensable linking of gene-rich contigs onto both physical and genetic maps.

## Conclusion

MSLL and HMPR technologies generate uniquely interesting sets of gene-rich sequences, offering significant benefits as part of an overall genome sequencing strategy. In particular, MSLL sequences access the DNA upstream and downstream of genes that are under-represented in other gene-enrichment survey sets. Each technique also generates paired reads, enhancing the assembly and linking of all gene-rich regions, and allowing the resulting contigs to be located on an FPC map.

Because the B73 cultivar of maize is now being fully sequenced using a BAC-by-BAC strategy [36,42], low-cost sequencing in this cultivar is no longer an urgent need, and none of the gene-rich sequencing strategies have been carried fully to completion. However, the gene-rich strategies can play a major role for other cultivars of maize and for the larger genomes (>5 Gb) that are found in the majority of flowering plants. Until such a project is attempted at a scale that tests the final steps in complete gene discovery and sequence analysis, the degree to which these tools can be applied across plant genomics will remain somewhat unclear.

Even in the context of BAC-by-BAC maize sequencing, the MSLL and HMPR sequences can play a valuable role in improving and correcting the assemblies, due to their use of paired reads, and their tendency to span highly repetitive and difficult-to-assemble genomic regions. Furthermore, placement of unmethylated sites and methylated blocks onto the genomic sequence provides an additional epigenetic level of genome annotation, indicating candidate sites for further analysis of the accuracy of gene discovery and for further study of poorly understood gene regulatory functions. A full display of MSLL/HMPR alignments to maize BACs, as well as alignments of other gene-rich sequences and repeat-database annotation, is provided at the Arizona Genomics Computational Lab [28]. The HC, MF, HMPR and MSLL clones can be ordered from the Arizona Genomics Institute [43].

## Methods

### HMPR library construction and end-sequencing

Nuclear DNA was extracted from immature ears of maize inbred B73 as previously described [14]. Partial digestions were performed as described in a previous study [25] with some modification. 20 μg of DNA were partially digested in 500 μL volumes with serially diluted restriction enzymes, *Hpa*II or *Hpy*CH4 IV (New England Biolabs), at 37°C for 30 min. Digestions were terminated by adding 50 μL of 0.5 M EDTA (pH 8.0). The digested fragments were dephosphorylated with shrimp alkaline phosphatase, then filled with G and C. The fragments were fractionated by agarose gel electrophoresis and the desired fragments (approximately 3–4 kb) were excised from the gel, recovered using the QIAEX II Gel Extraction Kit (QIAGEN Sciences), A-tailed with *Taq *polymerase, and inserted into pCR4TOPO using the Invitrogen TA cloning system. The constructed plasmids were electroporated into ElectroMax DH10B competent cells (Invitrogen/Life Technologies). Sequencing of HMPR clones was performed as previously described [25] except that reaction products were run on Applied Biosystems 3730XL sequencers.

### MSLL BAC library construction and end-sequencing

Megabase DNA in agarose plugs was prepared from young leaves or immature ears of maize inbred B73, digested overnight with *Sal*I or *Hpa*II, and separated on pulsed field electrophoresis gels following the standard protocol [44]. After two size selections, DNA fractions of approximately 20–35 kb, 35–60 kb, 60–100 kb and >100 kb were recovered. DNA fragments were electroeluted before use. Because the available BAC vectors do not contain unique sites for cloning *Sal*I or *Hpa*II restriction fragments, *Hin*dIII-*Sal*I and *Hin*dIII-*Hpa*II adaptors were used. Three oligos, oligo *Hin*dIII (5'-pAGCTGGTTCCCCTTGG-3'), oligo *Sal*I (5'-TCGACCAAGGGGAACC-3') and oligo *Hpa*II (5'-CGCCAAGGGGAACC-3') were synthesized. *Hin*dIII-*Sal*I and *Hin*dIII-*Hpa*II adaptors were prepared by mixing oligo *Hin*dIII with oligo *Sal*I and oligo *Hin*dIII with oligo *Hpa*II in TE buffer (10 mM Tris, 1 mM EDTA, pH8.0) at 250 mM each, respectively, and incubated at 65°C for 15 min, 37°C for 30 min and room temperature for >30 min. The BAC vector pAGIBAC1 was digested with *Hin*dIII and the digests were extracted with phenol/chloroform, precipitated with ethanol and ligated with each adaptor at 16°C overnight in the following reaction: 65 μl of H_2_O to resuspend each 15 μg of pAGIBAC1 *Hin*dIII digests, 20 μl of adaptor, 10 μl of 10× T4 DNA ligation buffer and 5 μl of T4 DNA ligase (Promega, 3 U/ml). The ligation products were separated by electrophoresis on 1% CHEF gels at 1–40 sec linear ramp, 6 volts/cm, 14°C in 0.5× TBE buffer for 16 hours. The 7.5 kb band was recovered. Library construction followed the standard protocol [44]. The 12–20 kb MSLL library was constructed using the TA cloning system and the clones were end sequenced in the same manner as the HMPR libraries described in this study. Sequencing of the MSLL BAC ends was performed as described previously [45].

### Alignment to BACs, ESTs, genes, AZMs, and organelles

Most sequences were aligned to the sequenced maize BACs using BLAT [46] with the following parameters: minScore = 400, minIdentity = 95, and maxIntron = 5. ESTs were aligned with maxIntron = 2000, and RescueMu used minScore = 200 (due to their shorter average length of 339 bp). The BLAT output alignments were further screened to require 98% sequence identity over at least 95% of the total query sequence. Paired hits were additionally restricted to lie within one sequence contig (unless the BAC was specified as having ordered sequence contigs) and to have opposite orientation for the two end alignments. Pairs were only accepted if each read hit uniquely within the whole BAC set. A number (248) of anomalously short MSLL alignments were discarded as likely artifacts of phase-I BAC assembly errors (the average size of the these alignments was 9.6 kb).

The repeat content of the BAC sequences was characterized by masking against the TIGR v4.0 maize repeat database [18], using RepeatMasker [47] with parameters xsmall, nolow, no_is, norna, ecrossmatch.

The sequences were also aligned to the maize EST contigs, which were assembled using the Program for Assembling and Viewing ESTs (PAVE, Soderlund et al., in preparation) with all ESTs from Genbank including the ESTs from the full length clones created for the maize FL-cDNA project [6]. EST alignments were performed using BLAT with minScore = 75, and then further screened to require 98% identity, and a minimum 100 bp match.

The sequences were aligned to 151 curated gene regions (B. Barbazuk, pers. comm.) using BLAT with parameters of minScore = 300 (200 for the RM), maxIntron = 5. Alignments were further screened to require 98% identity and a 300 bp match (200 for RM). The alignment was required to be a complete embedding of the query to within 3 bp of each end, or to be a consistent end-overlap, extending to within 3 bp of an endpoint on both query and target (for example, it is not consistent for part of a query to embed to the middle of a target).

The sequences were also aligned to maize mitochondrial (Genbank: AY506529) and chloroplast (Genbank: NC_001666.2) genomic sequences using parameter minScore = 200, maxIntron = 5. Alignments were further screened for 98% identity and at least 95% coverage of the query sequence. Since the organelle genomes are circular, the alignments were also run against the organelle sequences with the break point shifted to a different location. In screening for potential chimeric clones, only clones with both reads >300 bp were used; from this set, organelle similarity was found for 1247 (3.4%) of MSLL and 1284 (6.9%) of HMPR. Out of these, 150 and 44 clones, respectively, had only one end aligning to the organelle, and were studied further as possible chimerics. Those for which the organellar end also aligned to the sequenced BACs (with parameters listed above) were discarded as presumed organelle-related DNA in the nuclear genome [29]; this accounted for 109 MSLL and 36 HMPR candidates. The remaining potential chimerics were checked for alignment of the second end to the organelle with relaxed parameters (no filtering of the BLAT hits above). If the second alignment was found, the clone was presumed to be non-chimeric, with the lower-stringency match reflecting either nuclear-incorporated organelle DNA or sequencing error in the organelle sequence. This test cleared an additional 31 candidates, leaving 16 presumed MSLL chimerics and 2 HMPR.

MSLL and HMPR sequences were masked against the TIGR v4.0 maize repeat database, using RepeatMasker with parameters xsmall, nolow, no_is, norna, ecrossmatch, qq, and then aligned to the TIGR AZM v5.0 assemblies [18]. The alignments were performed with BLAT using parameters minScore = 100, mask = lower -qMask = lower and filtered to require 98% identity, and either 95% match of the entire query or target sequence or an overlap of at least 100 bp extending to within 3 bp of the end of both query and target (a consistent end-overlap, as described above for the gene alignments).

Self-alignments of MSLL and HMPR libraries were performed using BLAT, requiring 98% identity across 95% of the shorter read length. For the pairing fraction measurement of MSLL clones, soft masking was used (BLAT parameters -mask = lower and -qMask = lower) to reduce spurious matches.

### Integration into the HICF FPC map

Six MSLL plates (La0009, Lb00011, La00012, Lf0001, Lh0001) were fingerprinted using the two-enzyme HICF technology previously described [30]. Peaks were scored automatically using thresholds equal to 10% of the highest peak in each color channel (in [30] the threshold was 25% of the 6th highest peak, but this had to be adjusted for the smaller MSLL clones, especially from the shorter libraries, because they often do not have six valid peaks in a color). Vector bands were removed and the fingerprints further screened to require at least half of the minimum expected band count for the size range of the clone library (using a conversion factor 1 band = 1.2 kb). Clones from the a and b libraries were required to have 14 bands; the f library required 25; and the h library required 41. With this criterion, 1022 successful fingerprints were generated, for an overall success rate of 53%. The maize HICF map was constructed using a two-enzyme variant of HICF [30] which is not compatible with the more widespread SNaPshot technique. Due to cost constraints, the reagents used were those remaining from the maize HICF production, and the age of the reagents most likely accounted for the lower success rate. However, it should be emphasized that the success rate of the fingerprinting itself is not material to the results of this paper, which hinge on the fraction of produced fingerprints that can be placed successfully on the physical map.

The successful fingerprints were placed onto the maize HICF map using the "Keyset → FPC" feature of FPC [26,27], with a cutoff at 1e-15. This feature automatically chooses the best contig (if any) in which to place the clone. As described in the text, 923 of the clones were placed successfully. The placements were checked using paired alignments to the sequenced maize BACs, generated as described above but screened with more relaxed standards, not requiring the paired end alignments to come from a single segment or to have the same orientation.

## Abbreviations

HMPR: hypomethylated partial restriction; MSLL: methyl-spanning linker library; HC: high-CoT; MF: methyl-filtration; RM: RescueMU; UF: unfiltered; BAC: bacterial artificial chromosome; BES: BAC-end sequence; EST: expressed sequence tag; AZM: assembled zea mays; MITE: miniature inverted-repeat transposable element; TIGR: The Institute for Genomic Research; UTR: untranslated region; GSS: genomic survey sequence; FPC: fingerprinted contigs; BLAT: BLAST-like alignment tool; HICF: high-information-content fingerprinting.

## Authors' contributions

JB conceived the study, and CS, JB, PM and RW are the principal investigators. CS and WN carried out data analysis and drafted the manuscript; JE, JB and PSM contributed to these efforts. ML and RH made the large-insert MSLL libraries; ML, JT, and NS made the remaining MSLL libraries. JA developed a vector for the MSLL cloning, although it was not used. DK performed the picking, arraying and condensation of the MSLL libraries. JB, JM, and ME developed the HMPR libraries. PSM supervised the HMPR end-sequencing; KC and HK carried out the MSLL BAC-end sequencing. AB and JM performed HICF fingerprinting of MSLL clones.

## References

[B1] International Rice Genome Sequencing Project (2005). The map-based sequence of the rice genome. Nature.

[B2] Du C, Swigonova Z, Messing J (2006). Retrotranspositions in orthologous regions of closely related grass species. BMC Evol Biol.

[B3] Bennetzen JL, Coleman C, Liu R, Ma J, Ramakrishna W (2004). Consistent over-estimation of gene number in complex plant genomes. Curr Opin Plant Biol.

[B4] Kikuchi S, Satoh K, Nagata T, Kawagashira N, Doi K, Kishimoto N, Yazaki J, Ishikawa M, Yamada H, Ooka H, Hotta I, Kojima K, Namiki T, Ohneda E, Yahagi W, Suzuki K, Li CJ, Ohtsuki K, Shishiki T, Otomo Y, Murakami K, Iida Y, Sugano S, Fujimura T, Suzuki Y, Tsunoda Y, Kurosaki T, Kodama T, Masuda H, Kobayashi M, Xie Q, Lu M, Narikawa R, Sugiyama A, Mizuno K, Yokomizo S, Niikura J, Ikeda R, Ishibiki J, Kawamata M, Yoshimura A, Miura J, Kusumegi T, Oka M, Ryu R, Ueda M, Matsubara K, Kawai J, Carninci P, Adachi J, Aizawa K, Arakawa T, Fukuda S, Hara A, Hashizume W, Hayatsu N, Imotani K, Ishii Y, Itoh M, Kagawa I, Kondo S, Konno H, Miyazaki A, Osato N, Ota Y, Saito R, Sasaki D, Sato K, Shibata K, Shinagawa A, Shiraki T, Yoshino M, Hayashizaki Y, Yasunishi A (2003). Collection, mapping, and annotation of over 28,000 cDNA clones from japonica rice. Science.

[B5] Seki M, Narusaka M, Kamiya A, Ishida J, Satou M, Sakurai T, Nakajima M, Enju A, Akiyama K, Oono Y, Muramatsu M, Hayashizaki Y, Kawai J, Carninci P, Itoh M, Ishii Y, Arakawa T, Shibata K, Shinagawa A, Shinozaki K (2002). Functional annotation of a full-length Arabidopsis cDNA collection. Science.

[B6] maizecdna The Maize Full-Length cDNA Project. http://www.maizecdna.org.

[B7] Messing J, Bharti AK, Karlowski WM, Gundlach H, Kim HR, Yu Y, Wei F, Fuks G, Soderlund CA, Mayer KF, Wing RA (2004). Sequence composition and genome organization of maize. Proc Natl Acad Sci USA.

[B8] Whitelaw CA, Barbazuk WB, Pertea G, Chan AP, Cheung F, Lee Y, Zheng L, van Heeringen S, Karamycheva S, Bennetzen JL, SanMiguel P, Lakey N, Bedell J, Yuan Y, Budiman MA, Resnick A, Van Aken S, Utterback T, Riedmuller S, Williams M, Feldblyum T, Schubert K, Beachy R, Fraser CM, Quackenbush J (2003). Enrichment of gene-coding sequences in maize by genome filtration. Science.

[B9] Yuan Y, SanMiguel PJ, Bennetzen JL (2003). High-Cot sequence analysis of the maize genome. Plant J.

[B10] Antequera F, Bird AP (1988). Unmethylated CpG islands associated with genes in higher plant DNA. Embo J.

[B11] Bennetzen JL, Schrick K, Springer PS, Brown WE, SanMiguel P (1994). Active maize genes are unmodified and flanked by diverse classes of modified, highly repetitive DNA. Genome.

[B12] Gruenbaum Y, Naveh-Many T, Cedar H, Razin A (1981). Sequence specificity of methylation in higher plant DNA. Nature.

[B13] Gruenbaum Y, Stein R, Cedar H, Razin A (1981). Methylation of CpG sequences in eukaryotic DNA. FEBS Lett.

[B14] Rabinowicz PD, Schutz K, Dedhia N, Yordan C, Parnell LD, Stein L, McCombie WR, Martienssen RA (1999). Differential methylation of genes and retrotransposons facilitates shotgun sequencing of the maize genome. Nat Genet.

[B15] Palmer LE, Rabinowicz PD, O'Shaughnessy AL, Balija VS, Nascimento LU, Dike S, de la Bastide M, Martienssen RA, McCombie WR (2003). Maize genome sequencing by methylation filtration. Science.

[B16] Raizada MN, Benito MI, Walbot V (2001). The MuDR transposon terminal inverted repeat contains a complex plant promoter directing distinct somatic and germinal programs. Plant J.

[B17] Cresse AD, Hulbert SH, Brown WE, Lucas JR, Bennetzen JL (1995). Mu1-related transposable elements of maize preferentially insert into low copy number DNA. Genetics.

[B18] Chan AP, Pertea G, Cheung F, Lee D, Zheng L, Whitelaw C, Pontaroli AC, SanMiguel P, Yuan Y, Bennetzen J, Barbazuk WB, Quackenbush J, Rabinowicz PD (2006). The TIGR Maize Database. Nucleic Acids Res.

[B19] Fernandes J, Dong Q, Schneider B, Morrow DJ, Nan GL, Brendel V, Walbot V (2004). Genome-wide mutagenesis of Zea mays L. using RescueMu transposons. Genome Biol.

[B20] Haberer G, Young S, Bharti AK, Gundlach H, Raymond C, Fuks G, Butler E, Wing RA, Rounsley S, Birren B, Nusbaum C, Mayer KF, Messing J (2005). Structure and architecture of the maize genome. Plant Physiol.

[B21] Clark RM, Linton E, Messing J, Doebley JF (2004). Pattern of diversity in the genomic region near the maize domestication gene tb1. Proc Natl Acad Sci USA.

[B22] Stam M, Belele C, Ramakrishna W, Dorweiler JE, Bennetzen JL, Chandler VL (2002). The regulatory regions required for B' paramutation and expression are located far upstream of the maize b1 transcribed sequences. Genetics.

[B23] Springer NM, Xu X, Barbazuk WB (2004). Utility of different gene enrichment approaches toward identifying and sequencing the maize gene space. Plant Physiol.

[B24] Yuan Y, SanMiguel PJ, Bennetzen JL (2002). Methylation-spanning linker libraries link gene-rich regions and identify epigenetic boundaries in Zea mays. Genome Res.

[B25] Emberton J, Ma J, Yuan Y, SanMiguel P, Bennetzen JL (2005). Gene enrichment in maize with hypomethylated partial restriction (HMPR) libraries. Genome Res.

[B26] Soderlund C, Humphray S, Dunham A, French L (2000). Contigs built with fingerprints, markers, and FPC V4.7. Genome Res.

[B27] Soderlund C, Longden I, Mott R (1997). FPC: a system for building contigs from restriction fingerprinted clones. Comput Appl Biosci.

[B28] Maize mini-BACS. http://www.agcol.arizona.edu/maize.

[B29] Noutsos C, Richly E, Leister D (2005). Generation and evolutionary fate of insertions of organelle DNA in the nuclear genomes of flowering plants. Genome Res.

[B30] Nelson WM, Bharti AK, Butler E, Wei F, Fuks G, Kim H, Wing RA, Messing J, Soderlund C (2005). Whole-genome validation of high-information-content fingerprinting. Plant Physiol.

[B31] Bruggmann R, Bharti AK, Gundlach H, Lai J, Young S, Pontaroli AC, Wei F, Haberer G, Fuks G, Du C, Raymond C, Estep MC, Liu R, Bennetzen JL, Chan AP, Rabinowicz PD, Quackenbush J, Barbazuk WB, Wing RA, Birren B, Nusbaum C, Rounsley S, Mayer KF, Messing J (2006). Uneven chromosome contraction and expansion in the maize genome. Genome Res.

[B32] Stein LD, Mungall C, Shu S, Caudy M, Mangone M, Day A, Nickerson E, Stajich JE, Harris TW, Arva A, Lewis S (2002). The generic genome browser: a building block for a model organism system database. Genome Res.

[B33] Solovyev VV, Salamov AA, Lawrence CB (1994). Predicting internal exons by oligonucleotide composition and discriminant analysis of spliceable open reading frames. Nucleic Acids Res.

[B34] Bennetzen JL (2007). Patterns in grass genome evolution. Curr Opin Plant Biol.

[B35] Messing J, Dooner HK (2006). Organization and variability of the maize genome. Curr Opin Plant Biol.

[B36] The Maize Genome Browser. http://www.maizesequence.org.

[B37] Liu R, Vitte C, Ma J, Mahama AA, Dhliwayo T, Lee M, Bennetzen JL (2007). A GeneTrek analysis of the maize genome. Proc Natl Acad Sci USA.

[B38] Tikhonov AP, Bennetzen JL, Avramova ZV (2000). Structural domains and matrix attachment regions along colinear chromosomal segments of maize and sorghum. Plant Cell.

[B39] Gendrel AV, Lippman Z, Yordan C, Colot V, Martienssen RA (2002). Dependence of heterochromatic histone H3 methylation patterns on the Arabidopsis gene DDM1. Science.

[B40] Zhang X, Yazaki J, Sundaresan A, Cokus S, Chan SW, Chen H, Henderson IR, Shinn P, Pellegrini M, Jacobsen SE, Ecker JR (2006). Genome-wide high-resolution mapping and functional analysis of DNA methylation in arabidopsis. Cell.

[B41] Soppe WJ, Jacobsen SE, Alonso-Blanco C, Jackson JP, Kakutani T, Koornneef M, Peeters AJ (2000). The late flowering phenotype of fwa mutants is caused by gain-of-function epigenetic alleles of a homeodomain gene. Mol Cell.

[B42] Wei F, Coe E, Nelson W, Bharti AK, Engler F, Butler E, Kim H, Goicoechea JL, Chen M, Lee S, Fuks G, Sanchez-Villeda H, Schroeder S, Fang Z, McMullen M, Davis G, Bowers JE, Paterson AH, Schaeffer M, Gardiner J, Cone K, Messing J, Soderlund C, Wing RA (2007). Physical and genetic structure of the maize genome reflects its complex evolutionary history. PLoS Genet.

[B43] Arizona Genomics Institute BAC Resource Center. http://www.genome.arizona.edu/orders.

[B44] Luo M, Wing RA (2003). An improved method for plant BAC library construction.

[B45] Kim H, San Miguel P, Nelson W, Collura K, Wissotski M, Walling JG, Kim JP, Jackson SA, Soderlund C, Wing RA (2007). Comparative physical mapping between Oryza sativa (AA genome type) and O. punctata (BB genome type). Genetics.

[B46] Kent WJ (2002). BLAT–the BLAST-like alignment tool. Genome Res.

[B47] RepeatMasker Home Page. http://www.repeatmasker.org.

[B48] Pampanwar V, Engler F, Hatfield J, Blundy S, Gupta G, Soderlund C (2005). FPC Web tools for rice, maize, and distribution. Plant Physiol.

